# mDurance: A Novel Mobile Health System to Support Trunk Endurance Assessment

**DOI:** 10.3390/s150613159

**Published:** 2015-06-05

**Authors:** Oresti Banos, Jose Antonio Moral-Munoz, Ignacio Diaz-Reyes, Manuel Arroyo-Morales, Miguel Damas, Enrique Herrera-Viedma, Choong Seon Hong, Sungyong Lee, Hector Pomares, Ignacio Rojas, Claudia Villalonga

**Affiliations:** 1 Department of Computer Engineering, Kyung Hee University, Yongin-si 446-701, Korea; E-Mail: cshong@khu.ac.kr; 2 Department of Library Science, University of Granada, Granada E18071, Spain; E-Mail: jamoral@ugr.es; 3 Department of Computer Architecture and Computer Technology, CITIC-UGR (Research Center on Information and Communications Technology), University of Granada, Granada E18071, Spain; E-Mails: nachodr@correo.ugr.es (I.D.-R.); mdamas@ugr.es (M.D.); hector@ugr.es (H.P.); cvillalonga@correo.ugr.es (C.V.); 4 Department of Physical Therapy, University of Granada, Granada E18071, Spain; E-Mail: marroyo@ugr.es; 5 Department of Computer Science and Artificial Intelligence, CITIC-UGR (Research Center on Information and Communications Technology), University of Granada, Granada E18071, Spain; E-Mail: viedma@decsai.ugr.es

**Keywords:** mobile health, digital health, physical conditioning, physical therapy, rehabilitation, trunk endurance, wearable inertial sensors, wearable electromyography sensors, mobile devices

## Abstract

Low back pain is the most prevalent musculoskeletal condition. This disorder constitutes one of the most common causes of disability worldwide, and as a result, it has a severe socioeconomic impact. Endurance tests are normally considered in low back pain rehabilitation practice to assess the muscle status. However, traditional procedures to evaluate these tests suffer from practical limitations, which potentially lead to inaccurate diagnoses. The use of digital technologies is considered here to facilitate the task of the expert and to increase the reliability and interpretability of the endurance tests. This work presents mDurance, a novel mobile health system aimed at supporting specialists in the functional assessment of trunk endurance by using wearable and mobile devices. The system employs a wearable inertial sensor to track the patient trunk posture, while portable electromyography sensors are used to seamlessly measure the electrical activity produced by the trunk muscles. The information registered by the sensors is processed and managed by a mobile application that facilitates the expert's normal routine, while reducing the impact of human errors and expediting the analysis of the test results. In order to show the potential of the mDurance system, a case study has been conducted. The results of this study prove the reliability of mDurance and further demonstrate that practitioners are certainly interested in the regular use of a system of this nature.

## 1. Introduction

Conservative treatments for low back pain (LBP) are gaining popularity due to the scientific evidence of their effectiveness. According to the Global Burden of Disease 2010 Study [1], LBP is the most common cause of disability. This disorder is also ranked sixth in terms of overall burden, with a global point prevalence of 9.4%. Furthermore, a recent study [2] has highlighted that the prevalence in the adult general population is approximately 12%, with a one-month prevalence of 23%, a one-year prevalence of 38% and a lifetime prevalence of more than 40%. Likewise, the prevalence of LBP among adolescents is also noteworthy, which is about 30% [3]. LBP has an enormous social and economic impact [4] and is a leading cause of absenteeism in all professions [5]. The growing interest of the scientific community in the study of LPB is also reflected in recent studies [6–8].

Pathophysiologically, LBP is associated with muscle weakness in the pelvic and lumbar region. This lack of endurance can lead to a poor lumbar-pelvic stability and, consequently, to the appearance of LBP [9]. General full-body exercises and encouraging the individual to stay active have been shown to be beneficial for preventing and dealing with chronic LBP [10]. However, in recent years, a major emphasis has been placed on the provision of more specifically directed exercises, which are aimed at targeting the muscles involved in low back stabilization. Therefore, more effective and efficient exercise programs can be developed. In order to establish goals, monitor the progress towards those goals and guide the prescription of specific exercises, a functional assessment of the trunk stabilization or endurance turns out to be utterly necessary [11]. Trunk muscle endurance assessment, normally referred to as trunk endurance assessment, consists of the evaluation of the muscular capacity of the individual's trunk. To determine the resistance of the trunk muscles, experts traditionally measure and annotate the observed time that the patient can hold a given posture during a test. Nevertheless, this form of evaluation is subject to potential errors, mainly posed by the subjectivity associated with the estimation of the test finalization and the effective measurement of the time elapsed during its execution [12].

Digital technologies can be used to cope with some of the limitations introduced by human errors during the practice of medical procedures. In fact, during the last few years, the use of devices and software in healthcare disciplines has become more common due to the constant technological improvement [13–18]. There are different factors attributable to the development of this type of system: the healthcare users demand for novel forms of treatment [19]; the globalization of health systems [20]; the need for the reduction of healthcare costs [21]; and the major advances in information and communication technologies [22]. Telehealth, eHealth, social health and health IT are some of the most prominent areas in which information and communication technologies are used to expedite and enhance healthcare procedures. Currently, at the forefront of the digital health revolution is the so-called mobile health (mHealth) [23], which refers to the practice of medicine and public health supported by mobile devices and applications. The interest in this domain has particularly boomed due to the growth of wearable and mobile technologies [24], as well as the intensive effort put by research institutions and companies into the development of digital health systems [25,26] and platforms [27–30].

In light of present challenges of physical rehabilitation and conditioning routines, as well as the potential of mHealth technologies, this work presents mDurance, a mobile health system intended to support experts in the functional assessment of trunk endurance by using wearable and mobile devices. The system has been defined to overcome some of the most relevant limitations faced by specialists during the course of endurance tests, such as the determination of the patient's initial posture, the estimation of the test duration and the measurement of the muscle fatigue. The mDurance system leverages the use of wearable inertial sensors to track the patient trunk posture and portable electromyography sensors to seamlessly measure the electrical activity produced by the trunk muscles. All of the information registered through these sensors is intelligently managed by a mobile application that builds on an mHealth framework developed in a previous work [31]. This app is devoted to facilitating the expert's normal routine, helping mitigate human errors and accelerating the analysis of the tests. The rest of the paper is structured as follows. Section 2 presents an overview of the state-of-the-art in mobile health applications for LBP. The fundamental principles of the trunk endurance assessment and most common tests are outlined in Section 3. The proposed mDurance system is described in Section 4. A preliminary case study is presented in Section 5, while final conclusions and remarks are summarized in Section 6.

## 2. Related Work

According to a survey performed in the U.K. [32], 75.5% of junior doctors and 79.8% of medical students own medical smartphone apps related to procedure documentation, disease diagnosis, clinical score and drug reference. The study highlights that the most frequently-used apps are devoted to detailing medication references, as well as disease diagnosis and management. In view of these findings, there is a clear motivation for the continuous development of medical apps. In fact, the high level of smartphone ownership and the more intuitive and user-friendly applications are compelling reasons suggesting that medical apps will offer a real opportunity to impact the efficiency of working practices and patient care. The market of medical applications is primarily led by Apple's iOS platform [33]; however, its use is tailored to a reduced and expensive catalog of devices. Alternatively, Android OS provides its users with a wider variety of devices of different prices and vendors in the reach of a broader audience, which is increasing its competitiveness in this domain [34,35].

**Figure 1:**
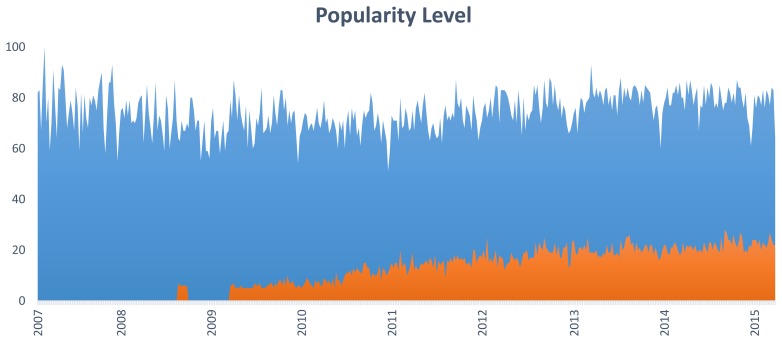
Interest over time in “low back pain” (blue chart) and “medical app” (orange chart) terms. Results obtained through Google Trends. The values, expressed in percentages, reflect the amount of searches that have been done for each term, relative to the total number of searches done on Google over time.

In our society, the utilization of the Internet to seek medical information has unarguably grown during recent years. Consequently, an analysis of the searches done over the Internet can help better understand the interest of people in medical tools and illnesses. Figure 1 particularly depicts the worldwide trends with respect to the search of “low back pain” and “medical app” concepts. “Low back pain” shows a constant popularity in people's searches over the last seven years, which might be related to the high prevalence of this disease and the demand for information regarding symptoms and potential treatments for this condition. The trend for “medical app” shows that people are increasingly interested in this sort of technology. It should be taken into account that these trends only refer to searches in English; thus, if other languages are considered, the popularity level could likely increase.

Several LBP-related apps can be found in the main application catalogs, *i.e.*, Google Play and Apple Store. The vast majority of apps are planned to promote exercises to prevent or relieve LBP. Furthermore, apps with informative or academic purposes and others focused on diagnosis are available. The number of apps to help alleviate LBP symptoms is especially elevated. Some examples are Stretch Away [36], Back Doctor [37], iREHAB [38], Prevent Back Pain [39], Yoga for Back Pain Relief [40], WebMD Pain Coach [41] and Upper & Lower Back Pain Relief [42]. These applications are mainly oriented to provide trunk exercise recommendations. They fundamentally consist of a database of image or video exercises, which are intended to guide the patient or person suffering from LBP on how to execute them. This category of apps is available for any sort of users, and normally, they do not take into account the potential diseases that may lead to LBP. The group of apps focused on providing patient or professional-oriented LBP information is also considerable. Some examples within this domain are Back Pain Guide [43], Back Pain Complete Guide [44], Back Pain: An Algorithmic Approach to Low Back Pain [45], Back Pain Causes And Cures [46] and Back Pain Nerve Chart [47]. This group of apps only offers information regarding the essentials of LBP, including causes, treatments or even descriptions of the back anatomy. Lastly, the apps dedicated to detect habits and postures that may lead to LBP are the least relevant in the marketplace at the moment. Some examples related to this group are PostureScreen Mobile [48], Clinical Pattern Recognition: Low Back Pain [49] and Virtual Diagnosis Spine [50]. The main purpose of these apps is to recognize LBP by requesting the users to provide information related to different LBP symptoms. Some of these applications also help customers identify different posture alterations.

A comprehensive search has been performed to find specific applications and systems to evaluate trunk endurance using traditional tests. To that end, the two major commercial app stores, *i.e.*, Apple App Store and Google Play, have been used. Furthermore, in order to determine the research relevance of this topic, a systematic search has also been performed through the Web of Science and Google Scholar portals. However, no relevant results have been obtained. In view of the search result, it seems that there is a clear opportunity for the development of applications and systems that may support specialists in trunk endurance assessment procedures.

## 3. Trunk Endurance Assessment

Different tests are available to assess the trunk endurance in people with or without LBP. These kinds of tests are performed by a specialist, and they normally consist of the measurement of the time a person can hold a specific posture involving the trunk muscles. During the execution of the test, the health professional has to control the patient position and decide when the test ends, according to some established termination criteria. The results obtained for a given patient help experts determine their status and muscular capacity, as well as their ability to hold a posture normally related to daily living activities.

**Figure 2:**
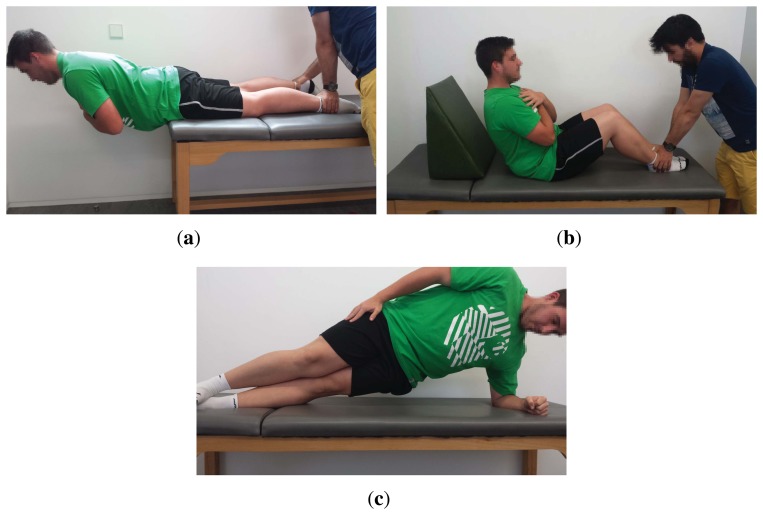
Trunk endurance assessment tests: (**a**) STEET; (**b**) TCSET; and (**c**) SBET.

Several functional trunk endurance tests to assess low back stabilization can be found in the literature [11,51]. The most widely-used ones are the static trunk extensor endurance test (STEET), also known as the Sorensen test [52], the trunk curl static endurance test (TCSET), also known as the trunk flexor endurance test [53], and the side bridge endurance test (SBET) [12] (see Figure 2). In the STEET, the subject has to maintain a horizontal unsupported posture, extending the upper body beyond the edge of the bench. In the TCSET, a curled position must be held with only the scapulae clearing the table. Finally, the SBET requires the individual to lie on their side while lifting the torso and thigh off the bench, such that the body weight is on the elbow and feet. It must be noted that two chances are given to the individual to execute the STEET, while evaluation of both left and right sides are considered as part of the SBET. A detailed description of each test, including posture, procedure and finalization criteria, is shown in Table 1.

According to the aforementioned scientific literature, there are some established normal endurance times for young and healthy men and women. The average endurance time for the STEET is established from 62 to 131 s. In the TCSET, the mean duration is 134 s, while for the SBET, it is approximately 84 s, with a standard deviation of 24.5 s. Using these endurance time references, experts can estimate whether a person has a good trunk endurance or not. Moreover, the relation among these values is found of relevance for the trunk endurance assessment. The ratios between flexor/extensor muscles and right/left sides are normally considered. These ratios show the equilibrium or disequilibrium between muscle groups. The ratio of trunk flexor to extensor endurance is 0.77 in normal conditions. The ratio of right side bridge to left side bridge endurance is normally 0.96. A reduced ratio of trunk flexor to extensor helps discriminate between LBP patients and healthy individuals, while a side to side difference greater than 0.05 suggests unbalanced endurance. The estimation of these reference values is explained in [11].

Based on the regular experience of specialists, practical limitations can be observed during the course of the realization and evaluation of endurance tests. First of all, it is generally accepted that the tester has an important responsibility while determining the different phases of the test. The estimation of the beginning and end of the tests is completely subject to the expert visual interpretation. In fact, specialists often report on the difficulties faced during the observation of the trunk angle variation and termination criteria [54]. Moreover, according to the nature of the tests [55,56], the specialist normally needs to control several aspects simultaneously, such as time, position and possible abnormalities during the test, which limits the routine uptake of the test. Finally, the results are mainly elaborated on the time recorded during the performance of the test, and that is the unique information to compare with in future tests. These drawbacks make the comparison of values measured by different testers and among sessions complex.

## 4. mDurance: A Novel System for Trunk Endurance Assessment

Taking into account the limitations of traditional trunk endurance assessment approaches, this work presents mDurance, an innovative system to support practitioners during regular trunk endurance assessment procedures. The mDurance system combines wearable sensors, capable of measuring physiological and biomechanical data, and mobile devices, dealing with the gathering, processing and persistence of the sensory data, as well as the visualization of health outcomes. Concretely, the system consists of a wearable inertial sensor to estimate the trunk position and an attachable electromyography sensor to measure the activity of the skeletal muscles of the trunk. All of the information generated by the sensors during the execution of the endurance tests is seamlessly and securely transmitted to a mobile application. This app was developed with some of the functionalities provided by a recent mobile health framework [31], here considered for the level of abstraction and agility offered to the developers. In the following, the key features of the mDurance system are thoroughly described.

### 4.1. Automatic Measurement of Trunk Posture

Determining the human trunk posture is of crucial importance to set the start of the endurance test, as well as to automate the identification of its completion. To do so, mDurance benefits from the use of an inertial measurement unit (IMU), which combines triaxial accelerometers, gyroscopes and magnetometers, enabling the measurement of the absolute attitudes or inclinations of the body part to which the sensor is fastened. This technology, extensively used in the navigation domain [57], has been exploited during recent years for body movement analysis [58–62]. Apart from their precision, these sensors are particularly interesting, since they are completely self-contained, thus not introducing constraints either in motion or any specific environment.

IMUs provide raw acceleration, angular rate and magnetic field data that need to be fused together to obtain a sole, optimal estimate of orientation. Diverse algorithms have been proposed in the literature to that end, including Kalman filters [63], least squares filters [64] or Gaussian particle filters [65], among many others [66,67]. The mDurance system particularly implements a recent technique, Madgwick's algorithm [68], which outperforms most existing approaches in terms of implementation complexity, sampling rate requirements and computational needs. This technique does not suffer from the well-known limitations of other solutions, like the singularity problem associated with the Euler angle representation (gimbal lock). Besides, this method also omits the use of computationally expensive trigonometric functions, making it more efficient and easier to implement for real-time purposes. Madgwick's algorithm employs acceleration, angular rate and magnetic field measurements to analytically derive, through an optimized gradient-descent method, a quaternion representation of motion [69]. Thus, the output of the algorithm is a quaternion, a compact vector in the form (*q*_1_, *q*_2_, *q*_3_, *q*_4_), which dynamically represents the orientation of the sensor. A detailed description of the foundations of the considered algorithm can be seen in [70].

Quaternions are frequently used in orientation estimation algorithms because of their numerical stability and computational efficiency However, this representation is difficult to interpret and visualize, since it defines a IR^4^ space that cannot be represented in a human-understandable three-dimensional view. Accordingly, a translation into Euler angles is performed here, after all of the calculations to estimate the quaternion are carried out. Euler angles represent the possible rotations around the three cardinal axes, namely yaw (*φ*), for the *X* axis, pitch (*θ*), for the *Y* axis, and roll (*φ*), for the *Z* axis. Given the estimated quaternion, the Euler angles can be simply obtained as follows:

(1) $$\varphi =\arctan\left(\frac{2\left(q_{1}q_{4}-q_{2}q_{3}\right)}{1-2\left(q_{1}^{2}+q_{2}^{2}\right)}\right)$$

(2) $$\theta =\arcsin\left(2\left(q_{1}q_{3}-q_{4}q_{1}\right)\right)$$

(3) $$\phi =\arctan\left(\frac{2\left(q_{1}q_{2}-q_{3}q_{4}\right)}{1-2\left(q_{2}^{2}+q_{3}^{2}\right)}\right)$$

### 4.2. Automatic Estimation of Muscle Fatigue

During the execution of the endurance tests, the muscles are normally subject to an important level of activity and stress. Having a continuous description of the evolution of this activity is of much clinical relevance to determine the muscle fatigue and potential physiological abnormalities [71]. As a consequence, mDurance incorporates a means to seamlessly monitor the electrical activity produced by the skeletal muscles. To that end, a wearable electromyography or EMG sensor is used. This sensor consists of a set of surface electrodes, which are attached to the skin of the body part to be monitored. The electrodes measure the potential difference between them, which is translated by the sensor into EMG signals. Experts usually focus on the analysis of the shape, size and frequency of the resulting electrical signals. However, there exist some well-known metrics that help categorize the level of the muscle fatigue. The root mean square (RMS), the average rectified value (ARV) and the maximum voluntary muscle contraction (MVC) are generally used as indices of muscle fatigue [72,73]. This information is of much interest to compare the evolution of the muscle strength among sessions, as well as to measure the effectiveness of potential treatments. Given the EMG signal and a time window or epoch of N samples, the RMS, ARV and MVC values can be calculated as follows:

(4) $$\text{RMS}=\sqrt{\frac{\overset{N}{\underset{k=1}{\text{∑}}}{\text{EMG}}^{2}\left(k\right)}{N}}$$

(5) $$\text{ARV}=\frac{\overset{N}{\underset{k=1}{\text{∑}}}\left|\text{EMG}\left(k\right)\right|}{N}$$

(6) MVC=max(EMG(k))

### 4.3. Sensor Setup and Application Description

One of the main aims of the mDurance system is to help experts assess, in a precise manner, the time invested by the patients during the execution of the trunk endurance test, as well as the amount of muscle fatigue experienced in that process. To attain the first objective, an IMU sensor is considered to determine when the test termination criterion is met, based on the principle presented in Section 4.1. For the second goal, an EMG sensor is used to continuously detect the electrical potential generated by the muscle cells in the course of the test, as explained in Section 4.2. Shimmer wearable sensors, concretely Version 2 for the EMG and Version 3 for the IMU, are employed, given the high reliability yielded by these commercial devices [74]. The default sampling rate configuration, *i.e.*, 51.2 Hz, is used for both sensors, since it proves to be enough for an accurate estimation of the trunk angle and EMG metrics.

**Figure 3:**
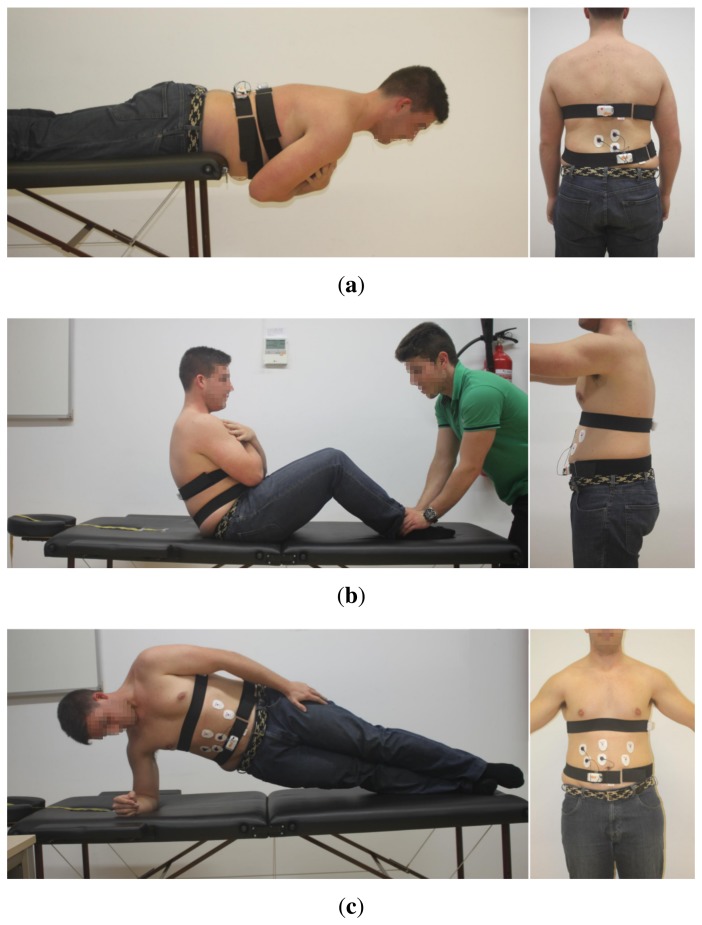
Sensor deployment for the (**a**) STEET, (**b**) TCSET and (**c**) SBET procedures.

Figure 3 shows the sensor deployment for each of the three trunk endurance tests supported by mDurance and described in Section 3. The sensors are located in convenient positions to ensure stability and comfortability, as well as an accurate measurement of both trunk angles and EMG values for each test. In the STEET and TCSET, the trunk angle is measured with respect to the coronal plane, while for the SBET, the reference corresponds to the sagittal plane. Accordingly, the IMU sensor is attached to the lumbar zone (D12-L1 vertebra) for the STEET and TCSET procedures and to the dorsal for the SBET. Taking into account the placement of the IMU sensor for each case and its local frame of reference orientation, the roll angle (*ϕ*) is used to represent the trunk angle in all tests. The EMG sensor is placed on the lumbar (erector spinae), abdominal (rectus abdominis) and external oblique parts for the STEET, TCSET and SBET, respectively. The electrodes are distributed to cover a sufficient muscle area. Both IMU and EMG sensors are safely and firmly fastened to each corresponding body part through ergonomic straps that ensure no misplacement of the sensors. Moreover, there is little room for errors during the placement of the devices, since the sensors must be positioned by the experts.

**Figure 4:**
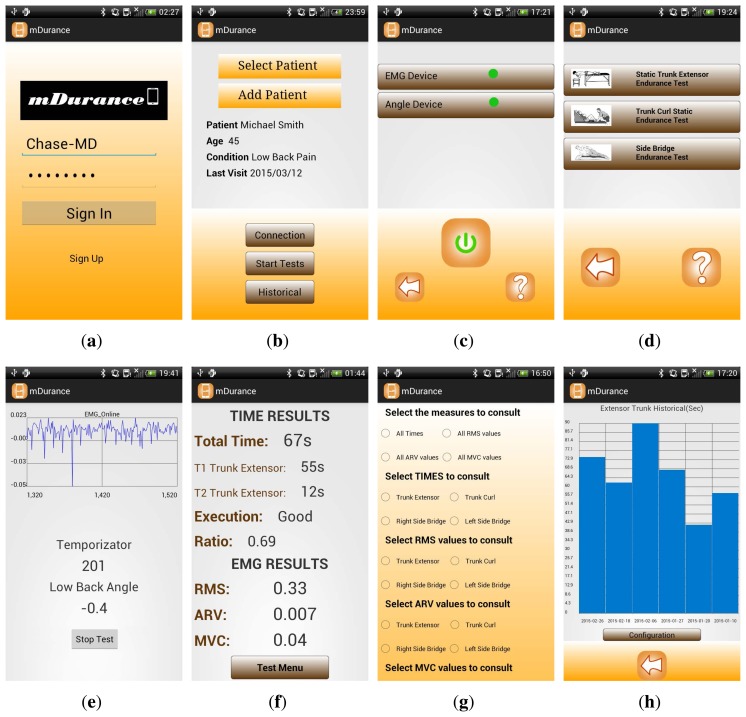
mDurance application snapshots: (**a**) login; (**b**) patient selection; (**c**) sensor connection; (**d**) endurance test selection; (**e**) test execution; (**f**) test results summary; (**g**) selection of historical attributes to be represented (part of); and (**h**) historical representation.

In the following, the mDurance application is described (Figure 4). For the first time use, the expert is requested to sign up with their personal information to register in the system. This information is used by mDurance to uniquely identify the specialist and also to preserve the patient's data collected by the system in a confidential and integral manner. Once an expert profile is created, the practitioner can log into the application contents by using their username and password (Figure 4a). Then, the expert is directed to a new screen, in which they can either select one of the existing patients in the system database or include a new one (Figure 4b). Personal information, such as name, age, height, weight, gender and possible health conditions, are requested when filling in a new patient registry. Upon selecting a patient, their more relevant personal information is presented to the expert for quick inspection, including the date of the last endurance session and particular conditions from which they suffer. Moreover, from this main screen, the expert can either initiate the connection with the wearable sensors, start the endurance tests or visualize the historical data collected during previous sessions.

The connection with the wearable sensors is performed by clicking on “Connection” (Figure 4b). During the very first configuration of the system, the sensors must be paired with the mobile device. To do so, the Bluetooth interface is activated, and both the mobile device and the Shimmer sensors are bound. After configuration, this one-time process is no longer required, unless the sensors are replaced. From then on, the expert can normally trigger the connection of the mobile and the wearable devices by pressing the power button (Figure 4c).

Once the sensors are connected and in order to proceed with the execution of the tests, the expert has to press “Start Tests” (Figure 4b). As a result, the specialist is directed to a new window in which the particular test to be performed can be chosen (Figure 4d). After selecting a test, another screen is displayed with the essential elements required by the expert to perform the test (Figure 4e). This includes a graph to visualize the recorded EMG signal at runtime; a timer to control the time left according to the maximum duration allowed for the realization of the test; and the trunk angle continuously measured by the system. The trunk angle is particularly useful for the expert to determine when the patient is correctly positioned. Then, once the specialist determines that the starting position is reached, the test can be initiated by clicking on the corresponding button. The angle measured at that moment is saved as a reference and used by the system to check whether the user exceeds the range defined for each test as part of the termination criteria. Thus, if the patient relaxes their posture more than ±10° in the STEET and SBET or ±30° in the TCSET, the test is automatically finished. The end-of-test is also attained when it lasts more than 240 s or when the expert explicitly considers that it should be finalized, for which the stop button can be used. After the test finalization, the expert can observe a summary of the results (Figure 4f). This includes the total duration of the test (sum of the two attempts for the STEET case), the endurance ratio and the RMS, ARV and MVC values. Furthermore, the session is categorized into “bad”, “good” and “perfect” based on the statistical overall duration of the patient, introduced in Section 3. Concretely, the ranges are bad = [0, 61*s*], good = [62, 131*s*] and perfect = [132, 240*s*] for the STEET; bad = [0, 133*s*], good = [132, 240*s*] for the TCSET; and bad = [0, 60*s*], good = [61, 108*s*] and perfect = [109, 240*s*] for the SBET.

Finally, the expert can inspect the patient's historical data by clicking on the “Historical” button (Figure 4b). This opens a new screen (Figure 4g), in which diverse types of representations can be selected, such as the time invested by the patient during the execution of the test and the muscle fatigue metrics. The results are depicted in a multi-date basis for the different past sessions registered in the system for the specific individual (Figure 4h).

### 4.4. App Implementation

**Figure 5:**
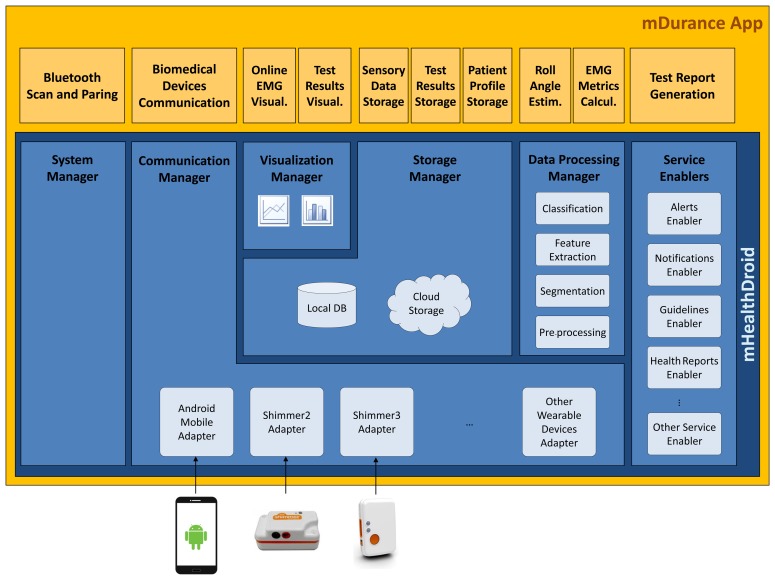
mDurance architecture building on mHealthDroid.

mDurance has been implemented using mHealthDroid [31], an open source framework devised to support the agile and easy development of mHealth applications on Android. mHealthDroid, which is released under the GNU General Public License Version 3 and available at [75], provides resource and communication abstraction, biomedical data acquisition, health knowledge extraction, persistent data storage, adaptive visualization, system management and value-added services. mHealthDroid has considerably facilitated the implementation of the mDurance core functionalities, such as the interface to the wearable sensors, the calculation of the test results, the persistent data storage and the visualization of the collected sensor information and historical test results (Figure 5).

The mDurance communication functionality relies on the mHealthDroid Communication Manager, which abstracts the underlying mobile and biomedical devices, makes the communication transparent to the application and provides a unified and interpretable data format. Concretely, the mHealthDroid Adapters for Shimmer2 (EMG) and Shimmer3 (IMU) wearable devices are used for these devices to communicate with the mobile phone and to map their data to the proprietary format. In this manner, the registered EMG potential and the triaxial acceleration, rate of turn and magnetic field samples are made available to the diverse components of the application. The security in the communication is handled through the Security Manager Protocol (SMP) of the Bluetooth Low Energy (BLE) protocol used for the data transfer transactions between the mobile device and the wearable sensors. mDurance performs a Bluetooth scan to detect available wearable devices and pairs them with the mobile phone. This functionality is implemented by using the mHealthDroid System Manager, which builds on the standard Android API [76].

One of the key features of mDurance is the estimation of the roll angle utilized to detect the trunk postures, the computation of the different endurance test times and the calculation of the RMS, ARV and MVC values based on the EMG signals. This functionality is developed with the mHealthDroid Data Processing Manager, which implements off-the-shelf signal processing techniques and data mining methods. This manager includes the algorithm considered for estimating the quaternion motion vector and its equivalent Euler angle representation, as well as the mathematical functions to extract the muscle fatigue features.

The sensory data collected during the endurance tests, the test results calculated by the mDurance core functionality, as well as the patient profile information are stored on a local database. The expert can register patients in the user database, including their name, age, gender and contact information, and update the personal information. The angle values and EMG collected during the endurance tests are buffered and periodically stored in the sensor table, in order to ensure efficiency and to facilitate the computation of some of the aforementioned metrics and results. Once the test is completed and the results are calculated, these are storedin the user table. The mDurance storage functionality builds on top of the mHealthDroid Storage Manager, which provides a high level of abstraction from the underlying storage technology and enables data persistence, both locally and remotely. In the current implementation, the mDurance app stores data locally on a SQLite database [77] deployed on the mobile phone SD card. However, the mHealthDroid Storage Manager also provides remote storage capabilities, which could enable the easy extension of the current mDurance application to store data on the cloud.

mDurance provides a graphical representation of online EMG values collected from the wearable device, as well as of the historical endurance test results, for example the test times and the calculated muscular fatigue values. Two types of graphical visualization are implemented using the mHealthDroid Visualization Manager, which supports diverse modes and ways to display data and builds on the open source library Graphview [78]. On the one hand, the data collected by the wearable EMG sensor and provided by the mHealthDroid Communication Manager are depicted on a line chart in an online fashion. On the other hand, the processed endurance test results, which are stored on the permanent storage and provided by the mHealthDroid Storage Manager, are represented on a bar diagram in an offline operation manner.

## 5. Evaluation

The proposed mDurance system has been designed taking into account some of the most important limitations faced by practitioners during the course of traditional trunk endurance assessment tests. Thus, in order to show the potential of this system, a preliminary analysis of its reliability and usability has been performed. To that end, ten volunteers, eight males and two females ranging from 21 to 37 years old, were recruited to be evaluated by three external physical therapists using both mDurance and traditional procedures. Before performing the evaluation, the volunteers were informed about the research aims, risks and benefits of participation, and they read and signed an informed consent form, previously approved by the University of Granada Ethical Committee. The procedures were executed sequentially, since a simultaneous evaluation cannot be performed. The reason is that the instructions given by the tester based on visual inspection, for example, finalizing the first attempt and starting the second chance in the STEET, can influence the normal flow of the decisions made through mDurance and *vice versa*. To procure the reproducibility of the tests, a rest time of more than one hour was considered to ensure the full recovery of the subjects in between the execution of both procedures. It is well-accepted in the physical therapy domain that endurance test results tend to be replicated, provided that the subject rests sufficiently in between tests and when these are performed in similar conditions [79]. The tests were explained to the subjects before performing the sessions, assuring the full understanding of their phases. Traditional sessions were performed as detailed in Section 3, while those involving the use of mDurance were carried out as described in Section 4. Accordingly, the execution was similar from the subject perspective, but the expert had to visually determine the start and end of each test and time it using a stopwatch in the traditional approach, while in the use of mDurance, these processes were automated.

**Figure 6:**
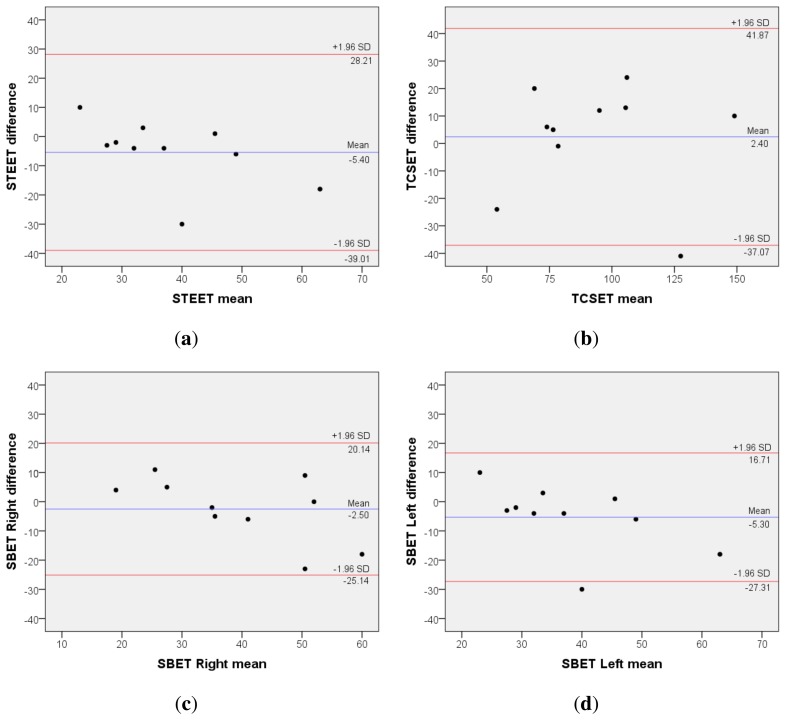
Agreement analysis between traditional trunk endurance assessment and mDurance through Bland-Altman plots: (**a**) STEET; (**b**) TCSET; (**c**) SBET right; and (**d**) SBET left. The mean of differences (*x̄*) is represented by a blue line, while the limits of agreement (*x̄* ± 1.96*σ_X_*) are depicted in red.

The first part of this evaluation aims at estimating the inter-rater reliability between the traditional trunk endurance assessment and mDurance. For that purpose, the results of the experiment, *i.e.*, the times measured for each individual, test and procedure are contrasted (Table 2). As can be observed, the results obtained through both traditional and mDurance methods are generally in line, which reflects the utility of the developed system. However, to support this observation, a formal statistical analysis is required. To that end, the intraclass correlation coefficient (ICC) (*ρ*) [80] Cronbach's *α* estimator [81] and the Bland–Altman “limits of agreements” statistic for continuous variables [82] are considered here. These statistics are widely utilized in the clinical domain to evaluate the agreement among two different instruments or two measurements techniques. One-way random effects ICC(*ρ*) and its confidence intervals (CI) are calculated for the inter-rater reliability trials. In accordance with previous studies [15,17], an ICC (*ρ*) value of less than 0.4 reflects poor inter-rater reliability; 0.4 to 0.75 represents fair to good reliability; and more than 0.75 is considered an excellent reliability. Similarly, a Cronbach's *α* statistic less than 0.5 is considered unacceptable; 0.5 to 0.6 is poor; 0.6 to 0.7 is questionable; 0.7 to 0.8 is acceptable; 0.8 to 0.9 is good; and a value greater than 0.9 represents an excellent reliability [83]. Finally, the Bland–Altman graphical technique is used to plot the differences between the measurements of the two procedures against their averages, which helps to better understand the agreement between both methods [84]. The results of the analysis are shown in Table 3 and Figure 6. SPSS Version 21.0 (IBM Corporation, Armonk, NY) is used for all of the statistical analyses.

The results of the statistical analysis prove a very high inter-rater reliability for all tests. More specifically, according to the ICC(*ρ*), the level of agreement between traditional assessment and mDurance can be categorized as excellent for all tests. Based on the Cronbach's *α* values, the reliability is excellent for the STEET, good for the TCSET and the SBET right and acceptable for the SBET left. The lower reliability values obtained for the SBET can be originated from multiple factors, such as the subject awareness or lack of concentration [53] and, especially, the level of fatigue experienced by the users, since this was the last test performed by them. The Bland–Altman graphical analysis shows a scattered distribution of the differences between both procedures that mainly falls within the 95% CI, thus not suggesting the presence of relevant disagreement. Though these results are very promising, a study including a higher number of subjects and applied to patients with LBP would be required to further confirm these findings.

The goal of the second part of this evaluation is to assess the usability and interest of mDurance according to the experts' opinions. Thus, after the realization of the tests, the three experts were asked to provide their impressions regarding the use of mDurance. First, they noted the practicality of the automatic angle measurement for initiating and finalizing the tests. In fact, they commented that the position adopted by the subjects through following the app guidance seemed to be more adequate than the one based on instructions from visual inspection. For example, a wedge is used in the TCSET to fix the initial position to an inclination of 60°, and then, this wood is pulled back ten centimeters before starting the test (Figure 2b). During the process of pulling back the wedge, individuals tend to relax the posture and bend the trunk more than required. This occurs while the expert is operating the wood; thus, the initial reference is usually not conserved. Conversely, specialists experienced more reliability when using mDurance, since they could just initiate the test whenever the appropriate angle was reached by the subject, as shown in the app. Likewise, the experts were truly impressed with the precision of the estimated angle and agreed that the finalization time was fairly determined. Furthermore, the real-time EMG representation was greatly appreciated, especially to observe the muscle contraction during the realization of the test. This feature, together with the calculation of RMS, ARV and MVC values were considered important assets of the system. The experts commented on the interest of having an automated log of time and muscle fatigue values to evaluate the patient improvement during their treatments or preventive interventions. In fact, they positively valued that all of the information is automatically storedin the system and that it can be retrieved and displayed at any time, even the data from prior sessions. They also considered this of much relevance for potentially constructing an evidence training program. Finally, the simplicity in the app usage and the friendliness of its interface were highlighted, as well. Indeed, this was considered during the development of the application, which seeks to attain ease of use and intuitiveness without sacrificing functionality.

Although experts did not report special negative comments, they mentioned that simpler guidelines should be provided along with the mDurance application to accelerate the understanding and usability of the whole system. During the first interaction with mDurance, they faced some troubles when connecting the sensors, which were nevertheless overcome after following the instructions given by the designers. Furthermore, they considered it desirable to share the data among diverse platforms, since the current version of the system limits its use to a single device. All of these valuable comments have been taken into account for future extension of this work.

**Figure 7:**
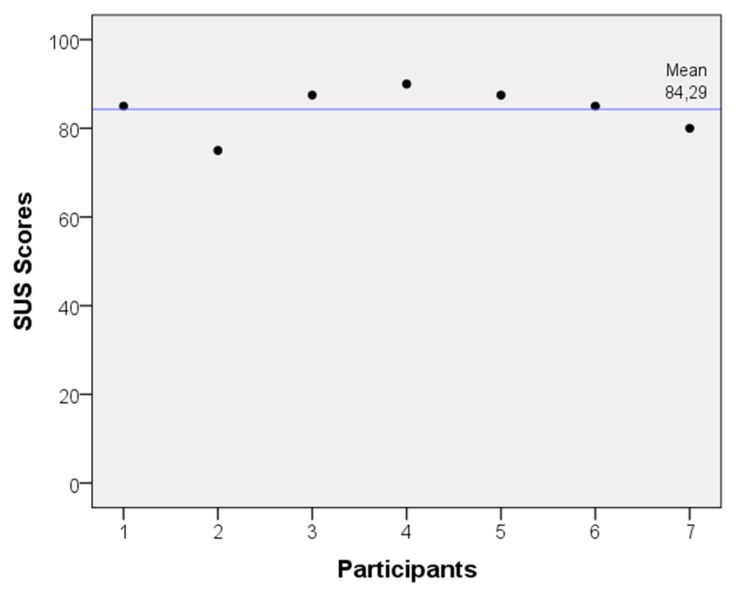
System Usability Scale (SUS) scores obtained from seven experts after using mDurance.

Apart from these qualitative outcomes, the usability of mDurance has also been evaluated in a more formal fashion by employing the System Usability Scale (SUS) [85,86]. This scale has nearly become an industry standard utilized to quantify the user experience with respect to diverse sorts of technologies. The SUS consists of ten statements that are scored by the user through a five-point scale anchored with “strongly disagree” and “strongly agree”. The SUS provides a point estimate of percentage usability. Scores above 70 are acceptable, while highly usable products score above 90. Scores below 50 indicate unacceptable low levels of usability [87]. For the evaluation, a total of seven independent experts were asked to use mDurance during a small trial. First, the experts were instructed on how to use mDurance, and after the finalization of the tests, they were requested to complete the questionnaire. The mean SUS score for the sample was 84.29 ± 5.15, as shown in Figure 7. SUS scores were generally high and indicated high levels of acceptability, ease of use and confidence when using mDurance. To further confirm the usability of mDurance, a study with a higher number of experts is needed, but preliminary findings seem to be favorable.

## 6. Conclusions

A spectacular proliferation of medical applications and systems has been observed during recent years; however, more significant contributions are still necessary to simplify, expedite and improve traditional health practices. In pathophysiology, trunk endurance assessment is a clear application area lacking appropriate tools. In fact, experts normally suffer from diverse kinds of limitations during the use of traditional procedures, such as difficulties in the precise estimation of the duration of the test, challenges in the evaluation of the muscle strength and other sorts of problems related to the subjective nature of each specialist assessment. Moreover, practitioners need to concentrate on measurement and annotation tasks, instead of focusing on the most relevant duties during the course of the test, like the analysis of the individual's behavior. To overcome these limitations, this work has presented mDurance, an innovative system that combines wearable inertial and electromyography sensors together with mobile devices for supporting a more accurate and rapid assessment of trunk endurance. The inertial sensors are used to continuously obtain the attitude of the trunk based on quaternions theory. This absolute trunk orientation helps experts determine when the user attains the correct posture to initiate the endurance test, as well as to automatically identify its finalization based on some established termination criteria. The electromyography sensor allows practitioners to observe the trunk muscles' activity during the execution of the tests, as well as the level of muscle fatigue experienced by the subject. All of the information is processed by a mobile application that was developed on a novel mHealth framework. The app significantly simplifies the routine of the expert and helps to manage the information collected from multiple individuals and sessions, which is considered of primal interest for tracking the evolution of the patients from visit to visit. An initial evaluation of the mDurance system has been performed to showcase the potential use of this system. The remarkable reliability and usability results demonstrate that mDurance can be accepted as a tool to measure trunk endurance. Finally, taking into account the high level of satisfaction shown by experts, the next steps include the use of mDurance on a large-scale clinical test bed, which is currently under development.

## Figures and Tables

**Table 1:** Trunk endurance tests description.

	**Static Trunk Extensor Endurance Test (STEET)**	**Trunk Curl Static Endurance Test (TCSET)**	**Side Bridge Endurance Test (SBET)**
*Patient position*	- Prone with the inguinal region/anterior superior iliac spine at the edge of the bench. - Arms at sides, ankles fixed (by strap or hands), holding horizontal position.	- Arms are folded across chest and back laid on a piece of wood to support the patient at a fixed angle of 60°. - Toes are anchored either with a strap or by the tester. - Both knees and hips are flexed 90°.	- The subject lies on one side supported by their pelvis, lower extremity and forearm. - The top leg is placed in front of the lower leg with both feet on the floor. - The upper arm is placed against the chest with the hand touching the anterior lower shoulder.
*Procedure*	- The patient maintains the horizontal position as long as possible. - Timing begins when the posture is horizontal and unsupported. - Subjects are verbally encouraged to hold this position as long as possible.	- The wood is pulled back 10 cm (4 in). - Timing starts when the initial posture is achieved. - The subject holds the isometric posture as long as possible.	- The pelvis is raised off the table as high as possible and held in line with the long axis of the body, supporting the weight between the feet and elbow. - Timing starts when the initial posture is achieved. - Subjects statically maintain this elevated position.
*Termination Criteria*	- The position is held up to a maximum of 240 s. - If the patient drops below the horizontal position more than 10° (an additional chance to regain it is given after the first attempt). - If the patient reports LBP or cramping in their legs, the test may be stopped.	- No specific time limitation, although generally considered a maximum of 240 s. - When any part of the subject's back touches the wood. This generally equals a drop of more than 30° with respect to the reference. - Significant LBP causes the test to be stopped.	- No specific time limitation, although generally considered a maximum of 240 s. - The subject is unable to lift their body up from the floor or drops their pelvis or thigh part way more than 10° and cannot raise it up to the start position again. - Significant LBP causes the test to be stopped.

**Table 2:** Case study results. BMI values are expressed in kg/m^2^ and the test duration in s.

**Patient ID**	*1*	*2*	*3*	*4*	*5*	*6*	*7*	*8*	*9*	*10*
**Age**	28	27	34	31	28	37	28	23	26	21
**BMI**	27.03	23.24	23.91	21.23	21.91	29.94	23.87	22.79	28.63	30.20
**STEET (T)**	43	56	59	121	104	48	98	123	59	75
**STEET (mD)**	32	59	108	123	99	60	105	117	52	85
**TCSET (T)**	42	79	107	112	101	79	118	78	77	154
**TCSET (mD)**	66	74	148	99	89	59	94	79	71	144
**SBET right (T)**	30	31	51	38	33	34	52	55	21	39
**SBET right (mD)**	25	20	69	44	38	36	52	46	17	62
**SBET left (T)**	26	28	54	46	35	30	35	46	28	25
**SBET left (mD)**	29	30	72	52	32	34	39	45	18	55

T, traditional method. mD, mDurance method.

**Table 3:** Inter-rater reliability between traditional trunk endurance assessment and mDurance.

**Variable**	**ICC (***ρ*)*	**CI 95% of ICC**^+^	**Cronbach's** *α*
**STEET**	0.92	0.68–0.98	0.92
**TCSET**	0.89	0.59–0.97	0.88
**SBET right**	0.84	0.39–0.96	0.83
**SBET left**	0.75	0.06–0.94	0.78

* ICC (*ρ*) was calculated using a one-way random model.

+ ICC indicates the intra-class correlation coefficient.

CI, confidence interval.
